# *In situ* targeting TEM8 via immune response and polypeptide recognition by wavelength-modulated surface plasmon resonance biosensor

**DOI:** 10.1038/srep20006

**Published:** 2016-01-29

**Authors:** Yimin Wang, Zewei Luo, Kunping Liu, Jie Wang, Yixiang Duan

**Affiliations:** 1 Research Center of Analytical Instrumentation, Key Laboratory of Bio-resource and Eco-environment, Ministry of Education, College of Life Science, Sichuan University, Chengdu 610065, PR China; 2 Faculty of biotechnology industry, Chengdu University, Chengdu, 610106, PR China; 3 School of Manufacturing Science and Engineering, Sichuan University, Chengdu, 610065, PR China

## Abstract

There is an increasing interest in real-time and *in situ* monitoring of living cell activities in life science and medicine. This paper reports a whole cell sensing protocol over the interface of Au film coupled in a wavelength-modulated surface plasmon resonance (WMSPR) biosensor. With dual parabolic mirrors integrated in the sensor, the compact and miniaturized instrument shows satisfactory refractive index sensitivity (2220 nm/RIU) and a high resolution of resonance wavelength shift of 0.3 nm to liquid samples. The affinity interactions between the biomarker of human tumor endothelial marker 8 (TEM8) and antibody (Ab) or specific polypeptide (PEP) were firstly introduced to WMSPR biosensor analysis. Both the interaction events of Ab-cell and PEP-cell over the Au film interface can be recognized by the sensor and the balance time of interactions is about 20 min. The concentration range of Ab for quantitative monitoring of the TEM8 expression on human colon carcinoma SW620 cells was investigated. The present low-cost and time-saving method provides a time resolution of binding specificity between Ab/PEP and TEM8 for real-time analysis of antigen on living tumor cell surface.

Tumor cell surface contains a lot of biomacromolecules such as glycoprotein, glycans, glycolipid and other kinds of proteins which include various biological information of cellular characteristics^1^. It is well known that abnormal expression patterns of biomacromolecules on cell membrane are usually associated with various of diseases, especially for cancers^2^,^3^. Thus an increasing kinds of diagnostic approaches have been developed to profile the expression of biomarkers on cytoplasmic membrane^4^,^5^. Among these biomarkers, tumor endothelial marker 8 (TEM8), a highly expressed cell surface membrane protein during tumor cell angiogenesis and migration^6^,^7^, was a newly identified conserved tumor marker both in mouse and human colon cancer tissue^8^,^9^,^10^. Due to its dual roles as anthrax toxin receptor 1 and cancer biomarker, targeting this antigen will help to develop an effective therapeutic strategy based on anti-angiogenesis^11^. However, by far we have little knowledge about the details of the binding behaviors between TEM8 and Ab/PEP at time resolution level, which was crucial for TEM8 targeted anti-cancer drug development based on anti-angiogenesis.

In current research advances, developing a selective fluorescent probe or isotope labeling reagent for imaging and spectral analysis is the mainstream direction to probe tumor tissue and cancer cells related biomarkers^12^,^13^. For example, a radioactive isotope or chemiluminescence dye labeled antibody can be used to imaging TEM8 expression *in vitro*^14^. However, compared to label-free method, these labeled imaging techniques can be only used at one time point, and may be harmful to human health and environment.

Over the past decade, many new biosensing techniques for *in-situ* and real time cellular analysis involved in bio-analytical chemistry^15^, such as the resonant waveguide grating biosensors (RWG)^16^, quartz crystal microbalance (QCM)^17^, light addressable potentiometric sensor(LAPS)^18^ and surface plasmon resonance (SPR) biosensor^19^, have been gradually established. Among these biosensing protocols, SPR, an optical sensing technology that characterizes the changes of refractive index resulting from binding events of the interface between the evanescent field and targets, is an emerging technique to characterize biomolecular interactions over the sensor surface^20^,^21^. For cytosensing, optical signal of SPR arises from the cellular response (e.g., cell mobility and viability) from extracellular stimulation, not directly from molecular binding. Since Giebel K.F. applied this technique in living cell analysis for the first time^22^, SPR has been widely used to characterize various cellular processes^23^, including cell morphology changes^24^, cellular response to environmental stress^25^, cell-protein interactions^26^,^27^,^28^ and other cellular activities^29^. This is mainly due to its advantages in label-free and real-time analysis of living cells, which are significantly important for cell based drug screening and evaluation.

By fixing the incident light near the resonance angle coupled in a prism, the resonance wavelength shift versus time of reflected light can be monitored by a spectrometer, which was named wavelength-modulated SPR (WMSPR)^30^,^31^. Comparing to the angle-modulated SPR, a WMSPR based instrument exhibited prospects of miniaturization and possibility to be used in remote analysis^32^. To date, however, wavelength-modulated SPR were widely applied to develop an immunosensor but rarely used in whole living cell biosensing^33^,^34^,^35^.

In the present study, a custom-designed and compact wavelength-modulated SPR setup was built using double parabolic mirrors. The refractive index sensitivity of the SPR sensor was evaluated. More importantly, Anti-TEM8 monoclonal antibody and a targeted sequence of polypeptide (PEP, KYNDRLPLYISNP, referred from reported literature^36^), which was able to specifically bind to TEM8 on membranes of human colon carcinoma cell line SW620, was utilized as a recognition element to target TEM8.

## Results

### The performance of WMSPR biosensor

**Figure 1:**
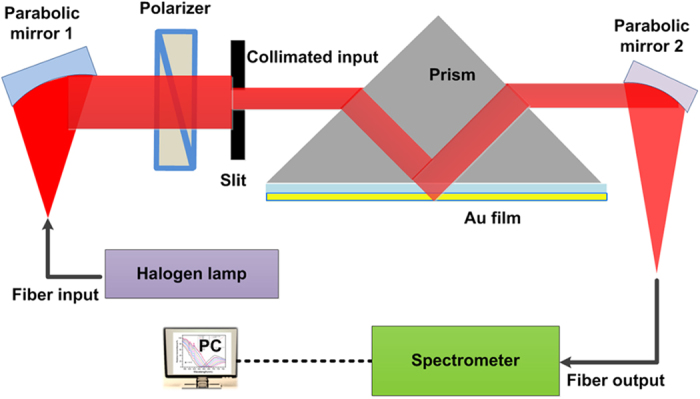
Schematic diagram of wavelength-modulated surface plasmon resonance (WMSPR) biosensor setup.

**Figure 2:**
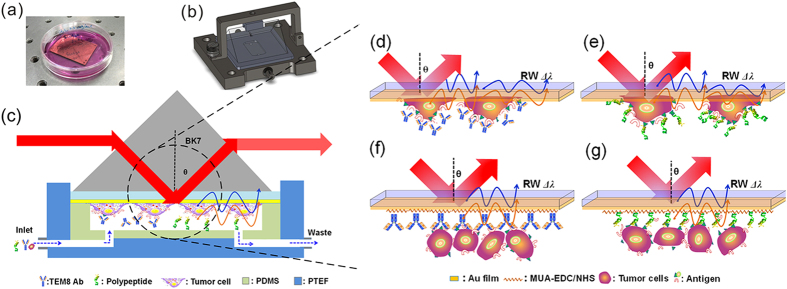
SPR sample flowing system and schematic representation of cell sensing on the Au film. Specific binding of Ab and PEP to TEM8 was introduced to SPR analysis. (**a**) The cell sensor chip in the culture dish. (**b**) 3D graphic diagram of SPR simple flowing system. (**c**) Schematic illustration of the sample flow system for Anti-TEM8 antibody (Ab) and polypeptide (PEP) based cytosensing. Cell coated sensor chip for Ab sensing (**d**) and PEP recognition (**e**). Ab (**f**) and PEP (**g**) coated sensor chips for cell capture.

The schematic diagram of WMSPR setup is shown in Fig. 1. It is worth mentioning that our self-designed SPR setup is compact and the sizes in three-dimension are 37.5 cm × 20 cm × 12.5 cm (see photograph of the setup in Supplementary Fig. S1 online). Figure 2 demonatrates the WMSPR sample flowing system and schematic representation of cell sensing testings on the Au film. The sensing system is facile to realize, due to two parabolic mirrors used to simplify the light path.

**Figure 3:**
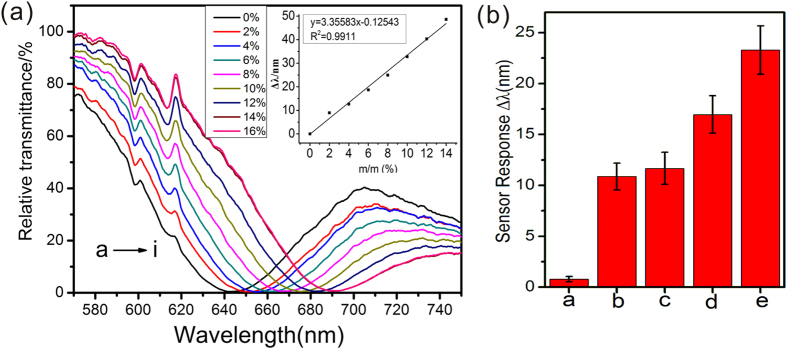
Refractive index sensitivity and sensor regeneration tests. SPR spectra of different concentrations of sucrose (**a**) versus wavelengths. Inserted graphs in (**a**), standard curve of resonance wavelength shifts in sucrose detection. (**b**) Human IgG immunoassay based SPR resonance wavelength shifts. (**a**) Water flowing, (**b**) MUA modification, (**c**) carboxyl activation with EDC/NHS, (**d**) immobilization of Anti-IgG antibody and (**e**) injection of 30 μg/mL IgG. All mean values and the standard error bars were obtained from three replicates of tests.

To demonstrate the sensitivity of the self-designed WMSPR sensor, the refractive index variations and immune sensing were conducted. In Fig. 3(a), with increasing concentrations (from 2% to 16%) of sucrose being injected into the sensing system, the resonance spectra from curve a to curve i show obvious red-shift and the corresponding resonance wavelength (RW) moved from 640 nm to 690 nm, which was attributed to the corresponding changes of refractive index of sucrose solution. The inserted graph in Fig. 3(a) shows the standard curve of resonance wavelength shifts of the resonance curves, which indicated that the net shifts of RW were well in proportion to the refractive index changes. Consistent results were also obtained in Fig. S2 (see Supplementary Fig. S2 online), which demonstrated the SPR detection of ethanol. The SPR sensing for immune response of human IgG was also investigated using anti-IgG antibody. In Fig. 3(c), the resonance wavelength shifts of five procedures in IgG detection were demonstrated. According to the defined refractive index sensitivity (RIS, RIS = Δλ/Δn, where Δλ and Δn were net shifts of resonance peak and refractive index, respectively) in reported work^37^, the standard curve of sucrose y(Δλ) = 3.36583*x*−0.12543, and the refractive index values in literature^38^, the relation between net shifts of peak wavelength (Δλ) and the refractive index of simple (*n*) could be described as Δλ = 2220.86*n*−2960.76. Considering RIS = Δλ/Δn, the RIS in our self-designed SPR sensor was ~2220 nm/RIU. Since the resolution of spectrometer using in the SPR sensing system was 0.3 nm, the presented sensor can detect the minimum change of refractive index at 1.5 × 10^−4^.

### Validate the expression of tumor endothelial marker 8

**Figure 4:**
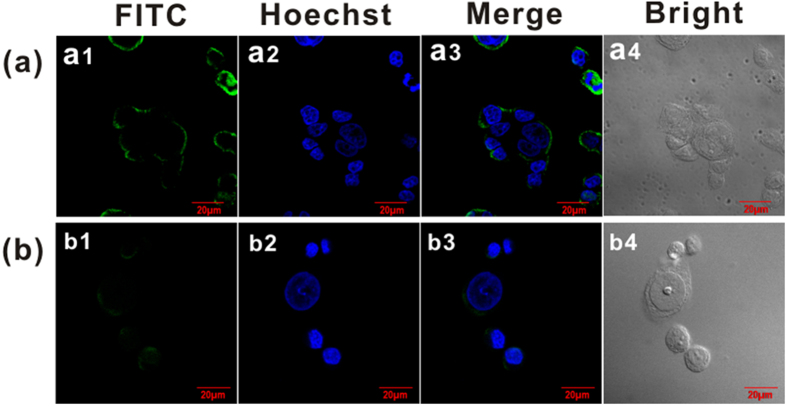
Cell immunofluorescence to evaluate the expression of TEM8. Human colon carcinoma cells of SW620 were incubated with Ab and FITC-conjugated goat anti-mouse IgG (secondary antibody) subsequently (**a**) or only incubated with FITC-labeled secondary antibody (negative control) (**b**). Green (a1 and b1) and blue (a2 and b2) channels show the locations of TEM8 and nucleus, respectively. The bright fields (a4 and b4) show the outline of tumor cells. Scale bar, 20 μm.

Indirect immunofluorescence assay is used to detect the antigen expression of tumor cells^39^. In Fig. 4, SW620 cells were incubated with Ab and FITC-conjugated goat anti-mouse IgG (FITC-IgG, also named secondary antibody) subsequently (a) or only incubated with FITC-labeled secondary antibody as the negative control (b). The green channel (FITC) shows the expression of TEM8 and the blue (Hoechst) indicates the locations of cell nucleus. As shown in the overlay images of a3 and b3, much stronger green fluorescence was found in a3 than that in b3, which demonstrates a high expression level of TEM8 in SW620 cells. Similar results were also obtained in another cancer cell line, MCF-7 (See details in Supplementary Fig. S3 online), which also confirmed that the anti-TEM8 antibody used throughout the experiments was able to specifically bind to cell surface.

### Anti-TEM8 antibody binding response

**Figure 5:**
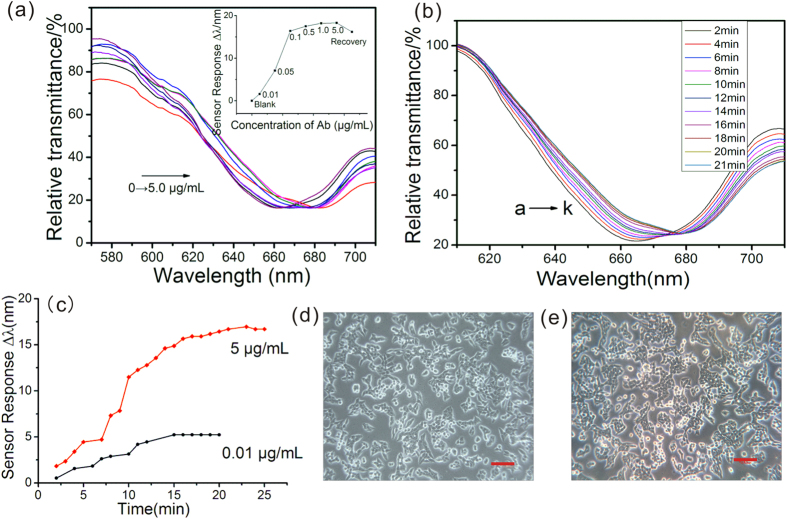
Anti-TEM8 antibody (Ab) based SPR cytosensing. (**a**) SPR resonance spectra of Ab with different concentrations. Inserted graph shows the net peak shifts of SPR wavelength against different concentrations of Ab. (**b**) Real-time recorded SPR cytosensing spectra at 5 μg/mL of Ab. (**c**) Resonance peak shifts versus reaction time at 5 μg/mL and 0.01 μg/mL of Ab. Cell morphology observations of SW620 under phase contrast microscopy before (**d**) and after (**e**) cell-Ab based SPR tests. Scale bar in (**d**,**e**) 50 μm.

Anti-TEM8 antibody (Ab) was used to excite the SPR response via binding SW620 cell over the sensor chip interface. After obtaining a steady resonance signal as the reference SPR signal by injecting HEPES buffer solution into the sampling system, different concentrations of Ab were introduced to the surface of sensor chip in sequence. As indicated in Fig. 5(a), comparing with the reference signal, the resonance wavelengths (RW) of Ab injection showed an evident change, which was attributed to the specific immune response between Ab and antigen epitope on the cell surface. However, as is well-known, interactions between cell membrane and Ab are sophisticated and random events. And the maximum penetration depth probing inside the sensing medium of SPR chip is ~ 200 nm^29^, thus only binding events occurred near the sensing chip surface on cell membrane can be detected. Therefore, the peak shifts of RW were not strictly proportional to the concentrations of Ab, which was in accordance with reported literature^40^,^41^. Finally, the cell sensor chip regeneration and desorption between cells and Ab was achieved by injecting 0.2 M Gly-HCl buffer (pH 2.6). It can be found in the graphic symbol of Fig. 5(a) (The recovery step) that even after 10 min of desorption, the RW cannot return to base line, which indicates high-affinity between cells and Ab.

On the other hand, to track the RW shifts in a time-resolved mode, the resonance spectra versus different reaction time were recorded and demonstrated in Fig. 5(b). It can be found that as the time went on, the RW gradually increased, which indicates increasing intensity of interactions in cell membrane mediated immune response. Similar results were also obtained by introducing 1 μg/mL Ab to the flow chamber (see Supplementary Fig. S4 online). Moreover, the resonance peak shifts versus reaction time in Fig. 5(c) demonstrated that the immune response achieved a balance after 20 min both at high (5 μg/mL) and low (0.01 μg/mL) concentrations of Ab, which was a short period for immune reaction. After all, Fig. 5(d,e) show the visual observations under phase contrast microscopy before biosensing and after SPR sensor regeneration, which displayed no evident changes of cell morphology.

### Polypeptide based specific recognition and SPR sensing

**Figure 6:**
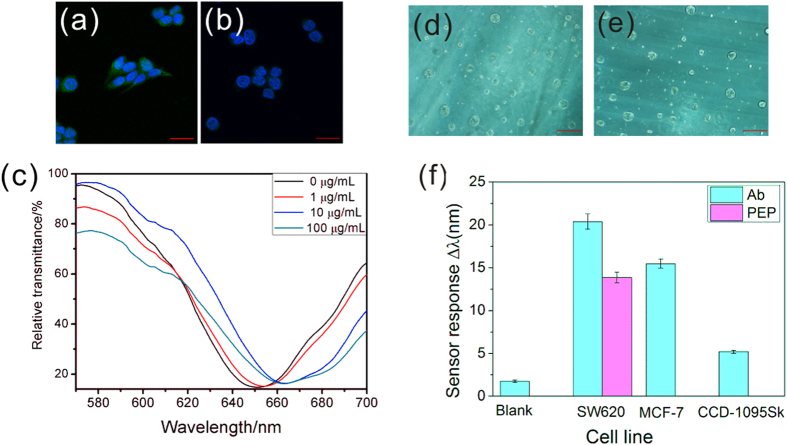
Comparison of polypeptide based fluorescence imaging and SPR cytosensing. Confocal imaging of fluorescence overlaps of SW620 cells treated with FITC-labeled polypeptide (**a**) and unlabeled polypeptide (**b**). Scale bar, 20 μm. Polypeptide (PEP) recognition based SPR cytosensing spectra (**c**). Photographs of Ab **(d**) and PEP (**e**) coated sensor chip surface after cell capture experiments. Scale bar in (**d**,**e**) 50 μm. (**f**) Resonant peak shifts of Ab and polypeptide (PEP) coated sensor chips for cell capture experiments based on three kinds of cell lines, Human colon carcinoma cell SW620, human breast carcinoma cell MCF-7 and human normal breast cell CCD-1095Sk. All mean values and the standard error bars were obtained from three replicates of tests.

The specific binding ability to TEM8 on SW620 cells of the synthetic polypeptides (PEP) used in the present work was also verified in a modified immunofluorescence-like assay. PEP-cell recognition based confocal imaging is demonstrated in Fig. 6. SW620 cells were treated with FITC-labeled PEP (a) and unlabeled PEP (b) as a negative control to exclude cellular auto-fluorescence. As shown in the merged images in Fig. 6(a,b), much stronger green fluorescence was found in (a) than that in (b), which indicates the high-affinity of the PEP and TEM8. Considering the facts discovered here, the monolayer cell sensing substrate on the Au film was also exposed to different concentrations of PEP. As displayed in Fig. 6(c), the RW shift observed at 10 μg/mL PEP was larger than that at 1 μg/mL but was nearly the same to 100 μg/mL. These results illustrate the facts that a stronger interaction occurred between PEP and TEM8 with the concentration increasing but the peak shifts reached its saturation concentration at 100 μg/mL.

### Binding specificity experiments

**Figure 7:**
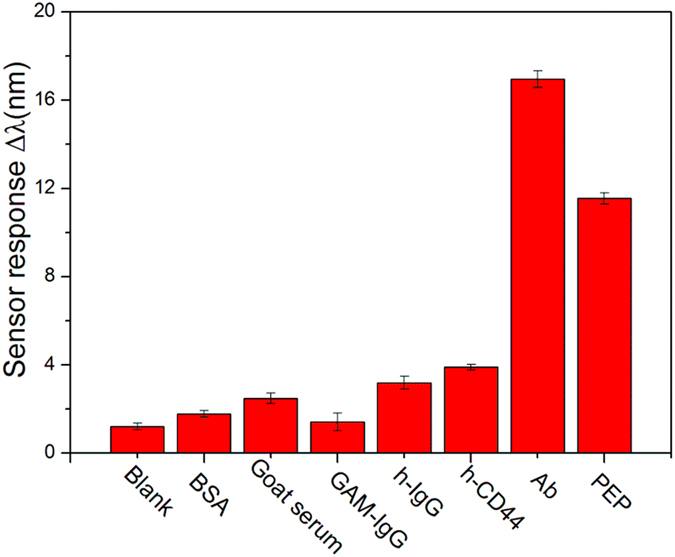
Specificity of antibody and peptide based SPR cytosensing. The 5 random selected proteins are 1% Bovine Serum Albumin (BSA), 1% goat serum, 5 μg/mL of goat anti-mouse immunoglobulin (GAM-IgG), human immunoglobulin (h-IgG) and human CD44 protein (h-CD44). HEPES buffer solution was injected as the blank control group. All mean values and the standard error bars were obtained from three replicates of tests.

Biological molecules can play roles of recognition elements to capture targeted cells, due to the specific binding between cells and analytes^42^,^43^. In order to explore the binding affinity of cell-Ab and cell-PEP on the Au film surface during SPR sensing, cell capture based SPR analysis was carried out. Micrographs obtained from scanning electron microscope (SEM) and fluorescence microscope determinate Ab and PEP coverage on the sensor chips (see Supplementary Fig. S5 online). In Fig. 6(d,e), direct observations of the testing area of the Au film surface were obtained under phase contrast microscope after cell capture based SPR assay. Although there is huge difference in size between cells and molecules, some cells still could be captured by Ab and PEP coated sensor chips and slight shifts of RW were found after 20 min of cell capture SPR experiments after non-specific adsorption had been excluded by Gly-HCl buffer solution (Fig. 6(f)). Considering the interface of biochip was simply modified for direct experiments in this work, more cells would be captured by amplification strategies to excite stronger SPR signal^44^,^45^. The sensor chip modification was optimized by comparing the cell capture capacity of the sensor chips using MPA and MUA. It can be seen that MUA significantly improved the cell binding ability of the sensor chip (see Supplementary Fig. S6 online). Cell capture experiments of SPR using human normal breast CCD-1095Sk cell line show much weaker sensor response than that of SW620 cells. Another human cancer cell line, MCF-7, which is also proved to be high-level expression of TEM8, also shows obvious sensor response (see Fig. 6(f) and Supplementary Fig. S6 online). All of these results indicate the sensor specificity for cancer cell analysis and are in accordance with cell immunofluorescence and polypeptide recognition based fluorescence imaging mentioned above. Additionally, the cytosensing specificity experiments of Ab and PEP in Fig. 7 also show that the cell sensor chip exhibits more evident resonance peak shifts response to Ab and PEP than other kinds of interfering proteins such as serum, antibodies and peptides.

## Discussion

The traditional labeled technique of immunofluorescence imaging (See cell immunofluorescence in Methods section) is sophisticated and time-consuming. Thus it was necessary to develop a label-free method in detection of TEM8 on cell surface. The main purpose of this paper is to demonstrate the feasibility to target living cell surface receptor of TEM8 using wavelength-modulated SPR biosensor. To the best of our knowledge, this is the first reported method combining wavelength-modulated SPR with living tumor cell sensing. In addition, the affinity interactions between the newly discovered biomarker of TEM8 and specific polypeptide were firstly introduced to SPR analysis.

The self-designed wavelength-modulated and Kretschmann prism configuration based SPR cytosensor using dual parabolic mirrors can simplify the light path of sensor setup. The biosensor shows an evident resonance wavelength response to target cell surface TEM8 both using Anti-TEM8 monoclonal antibody and specific sequence of polypeptide. Cancer cell lines with different expression levels of TEM8 can be distinguished by WMSPR analysis. For TEM8 detection on SW620 cell surface, the binding events between Ab and biomarker of TEM8 can induce the intracellular signaling pathways downstream this membrane protein and contribute to the refractive index changes close to the evanescence field. The concentration range of Ab for quantitative monitoring of the biomarker expression is 0.01~0.1 μg/mL (inserted in Fig. 5(a)). Concentration of Ab that higher than 0.5 μg/mL is unnecessary. The balance time of interactions between tumor cells and TEM8 antibodies was about 20 min and the minimum concentration of specific polypeptide that could be identified by tumor cells is 1 μg/mL, indicating that TEM8 was a promising target for anti-angiogenesis based anti-cancer drug development.

The present sensitivity of sensor can meet requirements of qualitative analysis of cancer cell, while the sensitivity of the sensor could be improved by an order of magnitude just by employing a better spectrometer (e.g., LTB ARYELLE 200 with spectral resolution ~0.084 nm, LTB, Berlin, Germany). Meanwhile, the cell binding capacity of the sensor chip was improved via reducing the sample flowing speed to 50 μL/min to avoid fluid shear stress that can be responsible for desorption of cells from sensor chip. In brief, after more experimental details being optimized in our following work, the developed WMSPR biosensor could be a promising approach for real-time qualitative and quantitative monitoring of the biomarker expression on tumor cell surface including other tumor surface antigens. Comparing with conventional techniques of immunofluorescence and western blotting, the intact living cells based *in situ* and label-free biosensor can provide a low-cost and time-saving detection of tumor cell. In addition, the polypeptide may act as a candidate of specific drug carrier in cancer therapy. The present sensor can also be adapted to other cellular process monitoring, which will benefit a better understanding to cellular behaviors related evaluations of antibody and drug screening.

## Methods

### Chemicals and cell lines

Poly-L-lysine (MW ≥ 300,000), Hoechst33342 and fluorescein isothiocyanate (FITC) were from Sigma-Aldrich, China. 3-Mercaptopropionic acid (MPA), 11-Mercaptoundecanoic acid (MUA) and ethanolamine (ETA) were obtained from Aladdin, China. 1-ethyl-3-(3-dimethyl aminopropyl) carbodiimide (EDC, purity 98.0%) and N-hydroxysuccinimide (NHS, purity 99.0%) were from Chengdu Ai Keda Chemical Technology Co., Ltd., China. Fetal bovine serum (FBS) was received from Biological Industries, Israel). 0.25% trypsin-EDTA, penicillin-streptomycin (100 units/ml and 100 μg/ml, respectively) were from Solarbio Co., Ltd., China). Dulbecco Modified Eagle Medium (DMEM, Hyclone) and 4-(2-hydroxyethyl)-1-piperazine ethanesulphonic acid (HEPES) were obtained from Aoke Biotechnology Co., Ltd., China. Mouse monoclonal antibody of Anti-TEM8 was purchased from Abcam (Clone number 200C1339). FITC-conjugated goat anti-mouse IgG as a secondary antibody was purchased from OriGene (Catalog No. TA130013). The synthetic FITC labeled and unlabeled polypeptide chains with sequence of KYNDRLPLYISNP (MW = 1592.83, purity >99%) were obtained from Shanghai Top-peptide Biotechnology Co., Ltd., China. Human colon carcinoma SW620 cell line, human breast carcinoma MCF-7 cell line and human normal breast CCD-1095Sk cell line were kindly provided by State Key Laboratory of Biotherapy, Sichuan University and Shanghai Cell Bank of Chinese Academy of Sciences. Other reagents are of analytical grade and sterile water was used throughout the whole experiment.

### Fabrication of SPR setup

In our custom-made wavelength-modulated SPR equipment, a white compound light beam emitted from a halogen tungsten lamp (SLS201/M, Thorlabs, USA) was collimated to parallel light by a reflective collimator, which was also named parabolic mirror (RC02SMA-P01, Thorlabs, USA). Then the light was polarized by a linear polarizer (LPVISE050-A) and passed through a Kretschmann configuration of BK7 prism based on total internal reflection to realize plasmon resonance. After that, the reflected light coupled from the prism was collected by another parabolic mirror (RC12SMA-P01, Thorlabs, USA) and connected to a fiber-optic spectrometer (AvaSpec-ULS2048, Avantes China) with a wavelength resolution of 0.3 nm ranging from 190 to 750 nm. The shifts of resonance wave excited by simple injection and molecular interactions via wavelength scanning mode were recorded and analyzed by the software of AvaSoft 7.7.

### Cell lines, sensor chip and simple flowing system preparation

SW620 and MCF-7 cells were cultured at 37 °C in a humidified atmosphere of 5% CO_2_ and 95% air in DMEM containing 10% of fetal bovine serum and 1% of penicillin-streptomycin, and maintained in a CO_2_ incubator (MCO-15AC, Sanyo, Japan). Cell growth status was observed under inverted phase contrast microscope (MI-12, Guangzhou Ming-Mei Technology, Co., Ltd., China). To prepare the cell sensor chip, a BK7 glass slide (35 mm × 25 mm × 0.7 mm) was cleaned with piranha (H_2_O_2_:H_2_SO_4_ = 1:3, V/V) and N_2_ flow drying. Then 2 nm of Cr adhesive layer and 48 nm of Au film were evaporated onto the glass substrate by magnetron sputtering deposition (Institute of Optics and Electronics, Chinese Academy of Sciences). The thickness of Au film was monitored using a surface profiler (XP-200, Ambios Technology, USA). After that, the chip was rinsed with deionized water and sterilized under ultraviolet irradiation for 30 min. Finally, the Au film was coated with poly-l-lysine solution (0.02 mg/mL) and immersed in media with SW620 in cell culture dish at a density of 2 × 10^5^ cells/mL for 24 h before SPR experiments (Fig. 2(a)). To match the Au film with BK7 prism, the glass substrate of the chip was coated with a thin layer of BK7 matching liquid (n = 1.5167, Cargille Laboratories, USA). The 3D image of self-designed flowing system for SPR was presented in Fig. 2(b). The volume of the flow chamber is about 200 μL (20 × 10 × 1 mm in dimension). In typical SPR sensing experiments, fluid simple in mobile phase was injected onto the sensor chip surface in the flow chamber (Fig. 2(c)).

### Refractive index sensitivity, sensor regeneration and IgG immunosensing

The refractive index sensitivity is a crucial parameter for evaluating the SPR sensor and it can be measured by monitoring the shifts of resonance wavelength. Herein, the increasing concentrations of micromolecule solution such as sucrose and ethanol were utilized to test the signal response to the change of refractive index on the sensor surface. Typically, the concentrations of sucrose and ethanol solutions above varying from 2% to 20% (w/w), and 10% to 80% (v/v) were injected to the flow chamber. The fitted linear standard curve was plotted to explore the linear range of the refractive index. Finally, the refractive index sensitivity (RIS) was defined as Δλ/Δn (Δn, in refractive index unit (RIU)). To evaluate the immune response of the sensor, human IgG SPR immunoassay was conducted. The Au film was incubated with 8 mM MUA solution at room temperature overnight and activated by 400 mM EDC and 100 mM NHS for 10 min. After washing with deionized water, the sensor chip was immersed in 30 μg/mL of Anti-IgG antibody and incubated at 37 °C for 30 min. The redundant carboxyl groups on the chip were blocked by 1 M ethanolamine (ETA) for 40 min to avoid non-specific reaction. Scanning electron microscope (SEM, JSM-7500 F JEOL, Japan) and fluorescence microscope (IX83, Olympus, Japan) was used to confirm Ab and PEP coverage on the sensor chips. The Anti-human IgG antibody coated chip was mounted under the prism and various concentrations of human IgG were subsequently injected into the sample chamber. The corresponding resonance spectra of each concentration were recorded after 20 min.

### Cell immunofluorescence and polypeptide fluorescence imaging

To validate the expression of TEM8 on SW620 and MCF-7 and the specific recognition to human colon carcinoma cells of polypeptides, immunofluorescence and fluorescence imaging were conducted as the following steps. Cell coated coverslip was fixed with 4% paraformaldehyde at 4 °C for 10 min and blocked with 5% normal goat serum at room temperature for 1 h, and then incubated overnight with 10 μg/mL anti-TEM8 antibody at 4 °C. After that, the slide was incubated with FITC-conjugated goat anti-mouse antibody (10 μg/mL) at room temperature for 30 min. For the polypeptide recognition and fluorescence imaging, cells were incubated with 5 μg/mL FITC-labeled polypeptides in the dark at room temperature for 30 min. At last, cells were stained with Hoechst33342 (10 μg/mL) at 37 °C for 10 min. Note that the coverslips were washed slightly by PBST (phosphate buffer containing 0.2% Tween-20) for 3 times after each step. Cell fluorescence was observed under confocal laser scanning microscopy (CLSM) (FV1000, Olympus, Japan).

### Living cell based SPR biosensing

Although two cell lines referred above both exhibited high expression of TEM8, in this paper we mainly focus our work on SW620 to demonstrate the feasibility to target this cell surface receptor using wavelength-modulated SPR biosensor. Thus SW620 cells were seeded on the Au film with a density of 2 × 10^5^ cells/mL and incubated in a culture dish for 48 h. When the monolayer cell confluence reached to 90%, the cellular sensor chip was mounted in the sample flow system. All experiments were conducted at room temperature (25 °C) using running buffer of HEPES solution (including 20 mM HEPES, 120 mM NaCl, 5.3 mM KCl, 0.8 mM MgSO_4_, 1.8 mM CaCl_2_ and 11.1 mM dextrose, pH 7.4) containing 5% FBS. For anti-TEM8 antibody (Ab) based cytosensing, various concentrations of Ab (including 0.1, 1.0 and 5 μg/mL) were injected into the simple cell to flow over the surface of the cells coated chip with a reaction time of 10 min. At last, the chip regeneration profiles were measured by injecting 0.2 M Gly-HCl buffer (pH 2.6) with a flowing time of 10 min. For comparative biosensing of PEP (including 1, 10 and 100 μg/mL), the protocol was similar to that of Ab assay. Note that the unbound analytes were removed by reinjection of buffer solution. (d) ~ (g) in Fig. 2 demonstrate the living cell based SPR biosensing procedures. Additionally, SPR cell sensor chip was visually observed under phase contrast microscopy before biosensing assay and after the sensor regeneration.

### Cell capture and sensor specificity evaluation

To validate the binding affinity of cell-Ab and cell-PEP, SPR sensing chip immobilized with anti-TEM8 antibodies or polypeptide were utilized to capture SW620 cells, respectively. The coating method was the same as the human IgG immunosensing of Methods part above. The peak shifts of resonance waves were recorded and the testing areas of the sensor chip surface were monitored by phase contrast microscope after three kinds of cell lines, SW620, MCF-7 and CCD-1095Sk cell capture of SPR analysis. Finally, the specificity of the sensor was evaluated. SPR sensor response of 5 random selected proteins (approximately equivalent in concentration) in the lab including: 1% Bovine Serum Albumin (BSA), 1% goat serum, 5 μg/mL of goat anti-mouse immunoglobulin (GAM-IgG), human immunoglobulin (h-IgG) and human CD44 protein (h-CD44) were compared with 5 μg/mL of Ab and PEP.

## Additional Information

**How to cite this article**: Wang, Y. *et al.* *In situ* targeting TEM8 via immune response and polypeptide recognition by wavelength-modulated surface plasmon resonance biosensor. *Sci. Rep.* **6**, 20006; doi: 10.1038/srep20006 (2016).

## Supplementary Material

Supplementary Information

## References

[b1] Springer T. A. Adhesion receptors of the immune system. Nature 346, 425–434 (1990). 1974032 10.1038/346425a0

[b2] Dalton W. S. & Friend S. H. Cancer biomarkers—an invitation to the table. Science 312, 1165–1168 (2006). 16728629 10.1126/science.1125948

[b3] Shao M. L., Bai H. J., Gou H. L., Xu J. J. & Chen H. Y. Cytosensing and evaluation of cell surface glycoprotein based on a biocompatible poly (diallydimethylammonium) doped poly (dimethylsiloxane) film. Langmuir 25, 3089–3095 (2009). 19437775 10.1021/la9000158

[b4] Park H. Y., Lee K. J., Lee S. J. & Yoon M. Y. Screening of peptides bound to breast cancer stem cell specific surface marker CD44 by phage display. Mol. Biotechnol. 51, 212–220 (2012). 21979823 10.1007/s12033-011-9458-7

[b5] Pan P. W. *et al.* Cell surface glycoprotein profiling of cancer cells based on bioorthogonal chemistry. Anal. Bioanal. Chem. 403, 1661–1670 (2012). 22526661 10.1007/s00216-012-5989-4

[b6] Croix B. S. *et al.* Genes expressed in human tumor endothelium. Science 289, 1197–1202 (2000). 10947988 10.1126/science.289.5482.1197

[b7] Bradley K. A., Mogridge J., Mourez M., Collier R. J. & Young J. A. Identification of the cellular receptor for anthrax toxin. Nature 414, 225–229 (2001). 11700562 10.1038/n35101999

[b8] Carson-Walter E. B. *et al.* Cell surface tumor endothelial markers are conserved in mice and humans. Cancer Res. 61, 6649–6655 (2001). 11559528

[b9] Rmali K. *et al.* Tumour endothelial marker 8 (TEM-8) in human colon cancer and its association with tumour progression. EJSO-Eur. J. Surg. Oncol. 30, 948–953 (2004). 10.1016/j.ejso.2004.07.023 15498639

[b10] Fernando S. & Fletcher B. S. Targeting tumor endothelial marker 8 in the tumor vasculature of colorectal carcinomas in mice. Cancer Res. 69, 5126–5132 (2009). 19528090 10.1158/0008-5472.CAN-09-0725

[b11] Cryan L. M. & Rogers M. S. Targeting the anthrax receptors, TEM-8 and CMG-2, for anti-angiogenic therapy. Front. Biosci. 16, 1574–1588 (2011). 10.2741/3806 PMC3066103 21196249

[b12] Yu Q. L., Zhan X. F., Liu K. P., Lv H. & Duan Y. X. Plasma-enhanced antibody immobilization for the development of a capillary-based carcinoembryonic antigen immunosensor using laser-induced fluorescence spectroscopy. Anal. Chem. 85, 4578–4585 (2013). 23547735 10.1021/ac400226n

[b13] Yu Q. L., Wang X. & Duan Y. X. Capillary-Based Three-Dimensional Immunosensor Assembly for High-Performance Detection of Carcinoembryonic Antigen Using Laser-Induced Fluorescence Spectrometry. Anal. Chem. 86, 1518–1524 (2014). 24417246 10.1021/ac402973n

[b14] Kuo F. *et al.* Immuno-PET Imaging of Tumor Endothelial Marker 8 (TEM8). Mol. Pharm. 11, 3996–4006 (2014). 24984190 10.1021/mp500056d PMC4224515

[b15] Merkoçi A. Nanoparticles-based strategies for DNA, protein and cell sensors. Biosens. Bioelectron. 26, 1164–1177 (2010). 20678915 10.1016/j.bios.2010.07.028

[b16] Ferrie A. M., Wu Q. & Fang Y. Resonant waveguide grating imager for live cell sensing. Appl. Phys. Lett. 97, 223704 (2010). 21203351 10.1063/1.3522894 PMC3009755

[b17] Zhou T., Marx K. A., Dewilde A. H., McIntosh D. & Braunhut S. J. Dynamic cell adhesion and viscoelastic signatures distinguish normal from malignant human mammary cells using quartz crystal microbalance. Anal. Biochem. 421, 164–171 (2012). 22119070 10.1016/j.ab.2011.10.052

[b18] Wu C. *et al.* Cellular impedance sensing combined with LAPS as a new means for real-time monitoring cell growth and metabolism. Sens. Actuators, A 199, 136–142 (2013).

[b19] Robelek R. Surface plasmon resonance sensors in cell biology: basics and application. Bioanal. Rev. 1, 57–72 (2009).

[b20] Homola J., Yee S. S. & Gauglitz G. Surface plasmon resonance sensors: review. Sens. Actuators, B 54, 3–15 (1999).

[b21] Mani R. J., Dye R. G., Snider T. A., Wang S. & Clinkenbeard K. D. Bi-cell surface plasmon resonance detection of aptamer mediated thrombin capture in serum. Biosens. Bioelectron. 26, 4832–4836 (2011). 21700444 10.1016/j.bios.2011.05.049

[b22] Giebel K. F. *et al.* Imaging of cell/substrate contacts of living cells with surface plasmon resonance microscopy. Biophys. J. 76, 509–516 (1999). 9876164 10.1016/s0006-3495(99)77219-x PMC1302541

[b23] Abadian P. N., Kelley C. P. & Goluch E. D. Cellular analysis and detection using surface plasmon resonance techniques. Anal. Chem. 86, 2799–2812 (2014). 24502446 10.1021/ac500135s

[b24] Robelek R. & Wegener J. Label-free and time-resolved measurements of cell volume changes by surface plasmon resonance (SPR) spectroscopy. Biosens. Bioelectron. 25, 1221–1224 (2010). 19818594 10.1016/j.bios.2009.09.016

[b25] Vala M., Robelek R., Bocková M., Wegener J. & Homola J. Real-time label-free monitoring of the cellular response to osmotic stress using conventional and long-range surface plasmons. Biosens. Bioelectron. 40, 417–421 (2013). 22863117 10.1016/j.bios.2012.07.020

[b26] Liu F. *et al.* Detection of EGFR on living human gastric cancer BGC823 cells using surface plasmon resonance phase sensing. Sens. Actuators, B 153, 398–403 (2011).

[b27] Liu C. *et al.* Live Cell Integrated Surface Plasmon Resonance Biosensing Approach to Mimic the Regulation of Angiogenic Switch upon Anti-Cancer Drug Exposure. Anal. Chem. 86, 7305–7310 (2014). 25005895 10.1021/ac402659j PMC4372114

[b28] Liu Y. & Cheng Q. Detection of membrane-binding proteins by surface plasmon resonance with an all-aqueous amplification scheme. Anal. Chem. 84, 3179–3186 (2012). 22439623 10.1021/ac203142n

[b29] Yang C. T., Méjard R., Griesser H. J., Bagnaninchi P. O. & Thierry B. Cellular Micromotion Monitored by Long-Range Surface Plasmon Resonance with Optical Fluctuation Analysis. Anal. Chem. 87, (2014). 10.1021/ac5031978 25495915

[b30] Wang S., Boussaad S. & Tao N. Surface plasmon resonance enhanced optical absorption spectroscopy for studying molecular adsorbates. Rev. Sci. Instrum. 72, 3055–3060 (2001).

[b31] Liu X. *et al.* Wavelength-modulation surface plasmon resonance sensor. Trac-Trends Anal. Chem. 24, 887–893 (2005).

[b32] Homola J. *et al.* Spectral surface plasmon resonance biosensor for detection of staphylococcal enterotoxin B in milk. Int. J. Food Microbiol. 75, 61–69 (2002). 11999118 10.1016/s0168-1605(02)00010-7

[b33] Sun Y. *et al.* Design and performances of immunoassay based on SPR biosensor with magnetic microbeads. Biosens. Bioelectron. 23, 473–478 (2007). 17764924 10.1016/j.bios.2007.06.016

[b34] Zhang J. *et al.* A novel surface plasmon resonance biosensor based on graphene oxide decorated with gold nanorod–antibody conjugates for determination of transferrin. Biosens. Bioelectron. 45, 230–236 (2013). 23500369 10.1016/j.bios.2013.02.008

[b35] Zhang J. *et al.* Preparation of graphene oxide-based surface plasmon resonance biosensor with Au bipyramid nanoparticles as sensitivity enhancer. Colloids Surf., B 116, 211–218 (2014). 10.1016/j.colsurfb.2014.01.003 24480068

[b36] Quan Q. *et al.* Imaging tumor endothelial marker 8 using an 18F-labeled peptide. Eur. J. Nucl. Med. Mol. Imaging 38, 1806–1815 (2011). 21814853 10.1007/s00259-011-1871-4 PMC3200564

[b37] Liu F. *et al.* Effects of nanoparticle size and cell type on high sensitivity cell detection using a localized surface plasmon resonance biosensor. Biosens. Bioelectron. 55, 141–148 (2014). 24373953 10.1016/j.bios.2013.11.075

[b38] Handbook C. Handbook of chemistry and physics. CRC Press LLC, Boca Raton, FL, USA, (2002).

[b39] Kanitakis J. Indirect immunofluorescence microscopy for the serological diagnosis of autoimmune blistering skin diseases: a review. Clin. Dermatol. 19, 614–621 (2001). 11604309 10.1016/s0738-081x(00)00180-2

[b40] Hiragun T. *et al.* Surface plasmon resonance-biosensor detects the diversity of responses against epidermal growth factor in various carcinoma cell lines. Biosens. Bioelectron. 32, 202–207 (2012). 22204782 10.1016/j.bios.2011.12.004

[b41] Yanase Y. *et al.* Detection of refractive index changes in individual living cells by means of surface plasmon resonance imaging. Biosens. Bioelectron. 26, 674–681 (2010). 20673712 10.1016/j.bios.2010.06.065

[b42] Li T. *et al.* Detection of breast cancer cells specially and accurately by an electrochemical method. Biosens. Bioelectron. 25, 2686–2689 (2010). 20537525 10.1016/j.bios.2010.05.004

[b43] Xue Y., Ding L., Lei J. & Ju H. A simple electrochemical lectin-probe for *in situ* homogeneous cytosensing and facile evaluation of cell surface glycan. Biosens. Bioelectron. 26, 169–174 (2010). 20591645 10.1016/j.bios.2010.06.010

[b44] Chen H. *et al.* Label-free surface plasmon resonance cytosensor for breast cancer cell detection based on nano-conjugation of monodisperse magnetic nanoparticle and folic acid. Sens. Actuators, B 201, 433–438 (2014).

[b45] Wang Y., Dostalek J. & Knoll W. Magnetic nanoparticle-enhanced biosensor based on grating-coupled surface plasmon resonance. Anal. Chem. 83, 6202–6207 (2011). 21711037 10.1021/ac200751s

