# Supplementary material for: In situ targeting TEM8 via immune response and polypeptide recognition by wavelength-modulated surface plasmon resonance biosensor
Source: Sci Rep. 2016 Jan 29;6:20006. doi: 10.1038/srep20006 (PMC4731803; doi:10.1038/srep20006)
Supplement: Supplementary Information [file srep20006-s1.pdf]

Supplementary Information

In situ targeting TEM8 via immune response and polypeptide recognition by wavelength-modulated surface plasmon resonance biosensor

Yimin Wang[a], Zewei Luo[a], Kunping Liu[a, b], Jie Wang[c], & Yixiang Duan[a, *]

[a] *Research Center of Analytical Instrumentation, Key Laboratory of Bio-resource and Eco-environment, Ministry of Education, College of Life Science, Sichuan University, Chengdu 610065, PR China.*

[b] *Faculty of biotechnology industry, Chengdu University, Chengdu, 610106, PR China.*

[c] *School of Manufacturing Science and Engineering, Sichuan University, Chengdu, 610065, PR China.*

*Correspondence should be addressed to Y. D. (yduan@scu.edu.cn)

Research Center of Analytical Instrumentation, College of Life Science, Sichuan University, Chengdu 610065, PR China.

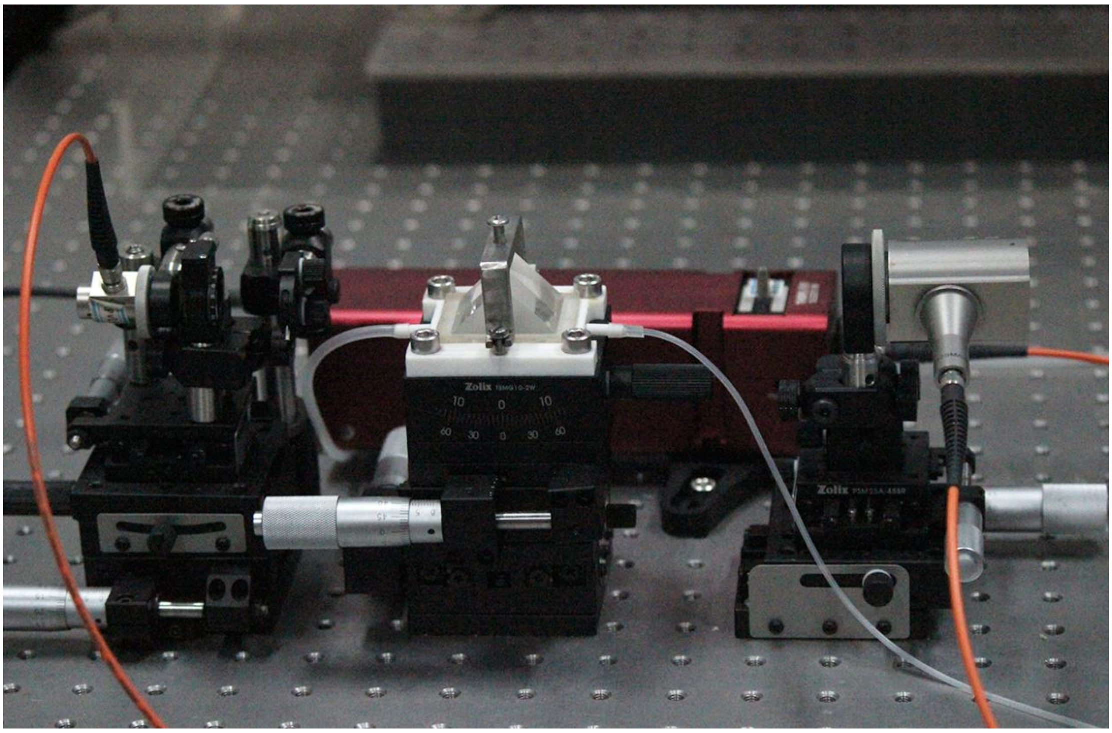

Supplementary Figure S1. Self-designed surface plasmon resonance (SPR) setup. The 3D size of whole instrument is about 37.5 cm×20 cm×12.5 cm.

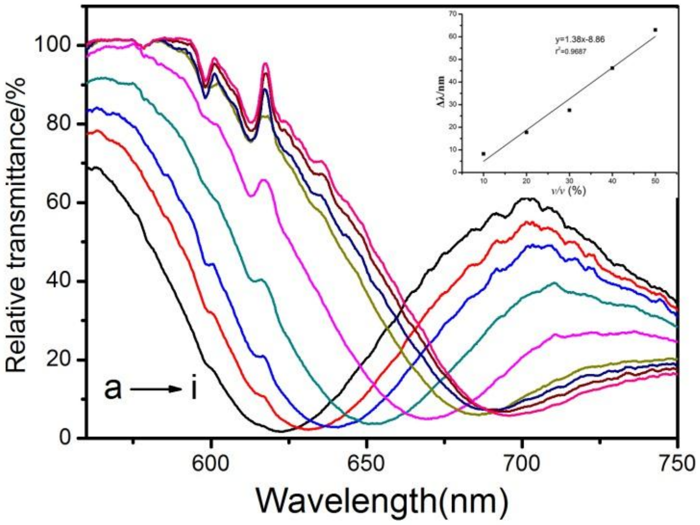

Supplementary Figure S2. SPR detection of different volume fractions of ethanol in water (from a to i) versus wavelength. Inserted graph shows the net shifts of resonance wavelength of different volume fractions.

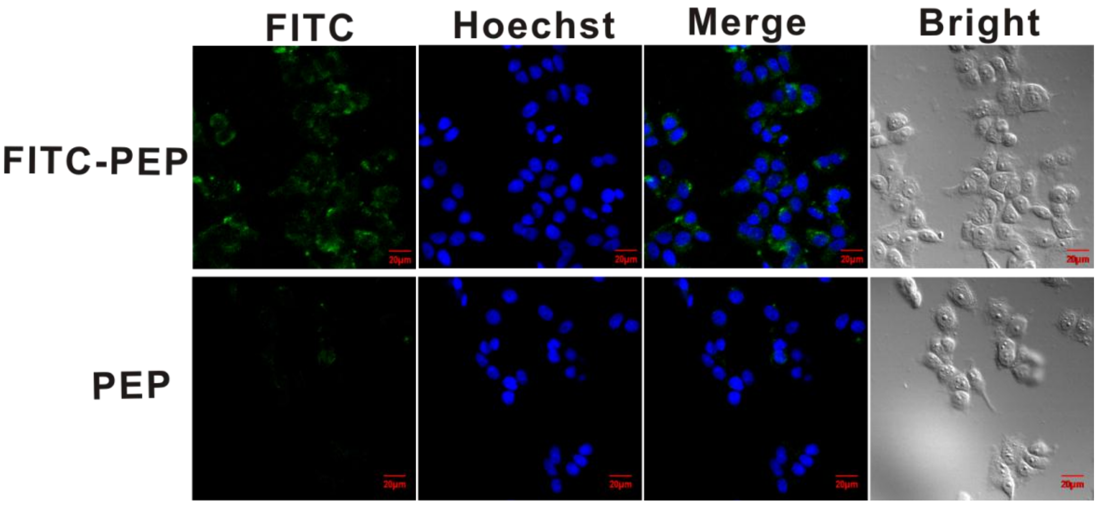

Supplementary Figure S3. Confocal laser scanning microscopy observations of MCF-7 cells (human breast carcinoma cell line) treated with FITC-labeled polypeptide (FITC-PEP) and unlabeled polypeptide (PEP). The negative control group shows only weak green fluorescence. Green and blue channels show the distribution of polypeptides and localizations of nucleus, respectively. The bright field indicates the outline of tumor cells. Scale bar, 20 μm.

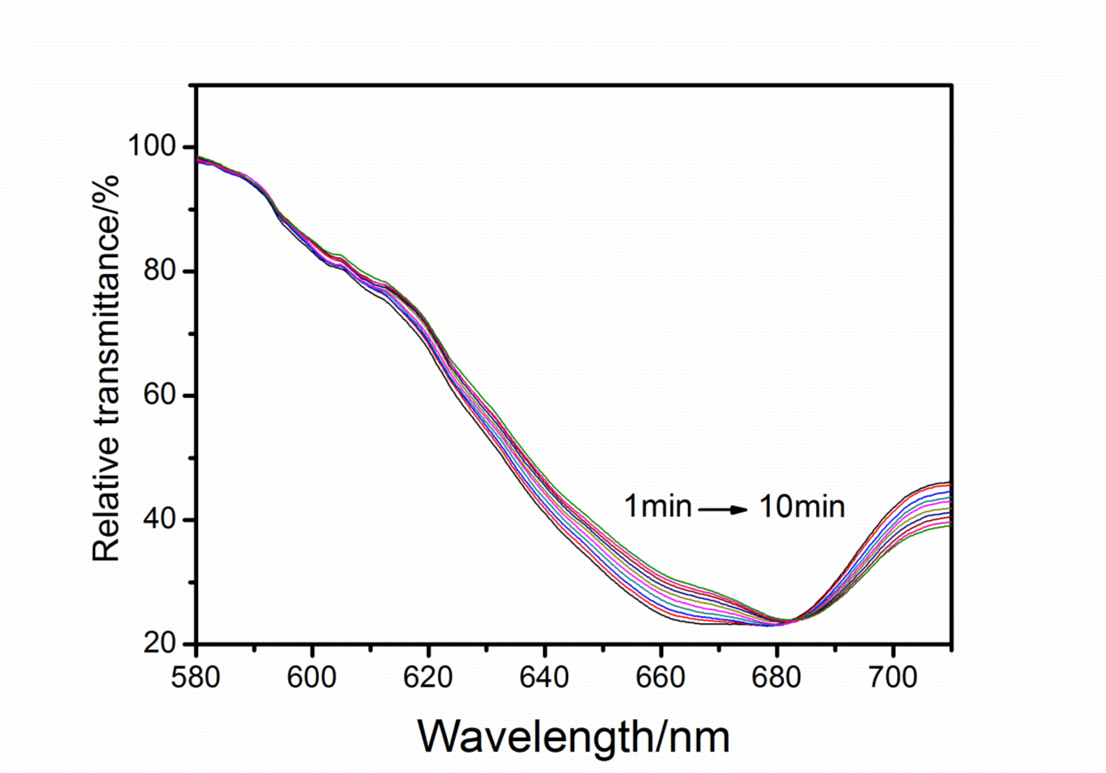

Supplementary Figure S4. Real time SPR cytosensing spectra upon injection of 0.1 μg/mL Anti-TEM8 antibody (Ab).

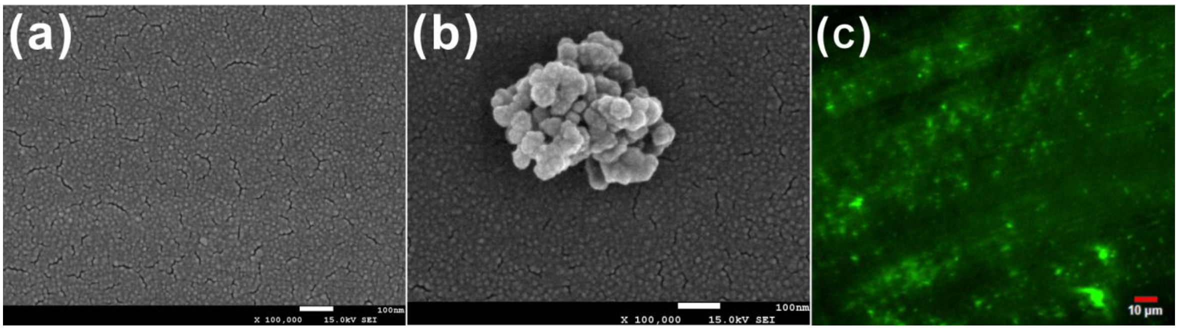

Supplementary Figure S5. Microscopic characterizations to determine Ab and PEP coverage on the sensor chips. SEM micrographs of MUA modified Au film for control (without antibody, (a)) or for Ab immobilization (with antibody, (b)). Scale bar, 100 nm (×100,000). (c) Fluorescence image of FITC-labeled PEP. Green spots show locations of PEP (or PEP aggregates) on the Au film.

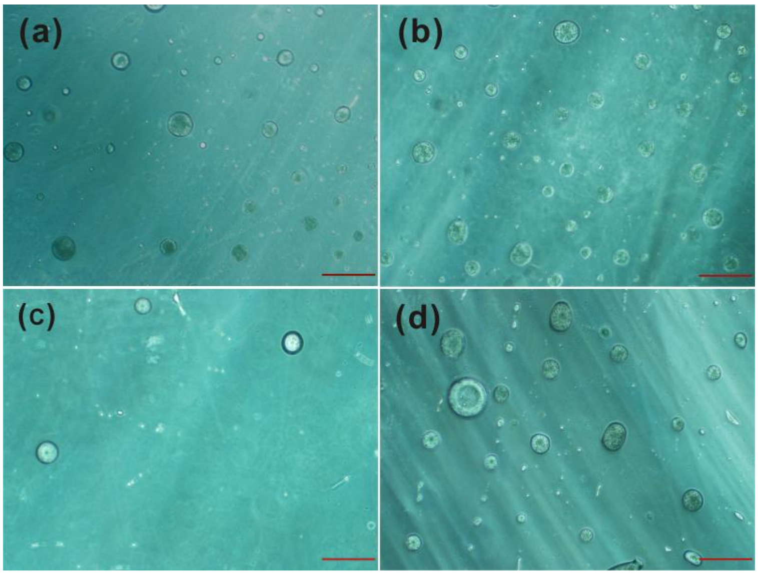

Supplementary Figure S6. Photographs under phase contrast microscope of Ab coated sensor chip surface after cell capture based SPR sensing tests. Ab immobilized with MPA (a) or MUA (b) on sensor chip for SW620 cell capture based SPR experiments. Ab immobilized with MUA for human normal breast CCD-1095Sk cell line (c) and human breast carcinoma MCF-7 cell line (d) based SPR experiments. Scale bar, 50 μm.
